# Precarious employment and mental health: the moderating role of household income and family type in Sweden

**DOI:** 10.1186/s12889-026-26259-x

**Published:** 2026-01-27

**Authors:** Signild Kvart, Lluís Mangot-Sala, Amanda Aronsson, Kathryn Badarin, Kim Bosmans, Virginia Gunn, Gun Johansson, Mireia Julià, Bertina Kreshpaj, Nuria Matilla-Santander, Fabrizio Mendez-Rivero, Emelie Thern, Per-Olof Östergren, Theo Bodin

**Affiliations:** 1 Unit of Occupational Medicine, Institute of Environmental Medicine, Karolinska Institutet, Solnavägen 4, Stockholm, 113 65 Sweden; 2 Centre for Global Health Inequalities Research (CHAIN), Department of Sociology and Political Science, Norwegian University of Science and Technology (NTNU), Edvard Bulls veg, Trondheim, 17049 Norway; 3 School of Sport, Nutrition and Allied Health Professions (SNAHP), Faculty of Health and Life Sciences, Oxford Brookes University, Oxford, OX3 0FL UK; 4 Department of Sociology, Brussels Institute for Social and Population Studies, Vrije Universiteit Brussel, Pleinlaan 2, Brussels, 1050 Belgium; 5 School of Nursing, Cape Breton University, 1250 Grand Lake Rd, Sydney, NS B1M 1A2 Canada; 6 Lawrence Bloomberg Faculty of Nursing, Toronto, ON Canada; 7 Social Determinants and Health Education Research Group (SDHEd), Hospital del Mar Research Institute, C/Dr. Aiguader, 80, Barcelona, 08003 Spain; 8 Hospital del Mar Nursing School (ESIHMar), Universitat Pompeu Fabra- affiliated, C/Dr. Aiguader, 80, Barcelona, 08003 Spain; 9 Complexity and Big Data Group, Department of Public Health, University of Copenhagen, Copenhagen, Denmark; 10 Barcelona Institute for Global Health (ISGlobal), Doctor Aiguader, 88, Barcelona, 08003 Spain; 11 Departamento de Bienestar y Salud, Universidad Católica del Uruguay, Av. 8 de Octubre 2738, Montevideo, 11600 Uruguay; 12 Social Medicine and Global Health, Department of Clinical Sciences in Malmö, Lund University, Box 117, Lund, SE 22100 Sweden; 13 Centre for Occupational and Environmental Medicine, Region Stockholm, Stockholm, Sweden

## Abstract

**Background:**

This study investigated the moderating role of household disposable income and family type on the association between precarious employment (PE) and diagnosed mental disorders.

**Methods:**

This longitudinal study used register data from the Swedish Work, Illness, and Labour-market Participation (SWIP) cohort. The study population included all individuals aged 27–65 who were employed in 2016 (*n* = 2,509,229). Precarious employment was measured using the Swedish Register-based Operationalization of Precarious Employment (SWE-ROPE 2.0), which captures employment insecurity, income inadequacy, and lack of rights and protection. Diagnosed mental disorders during 2017–2019 were identified through national inpatient, outpatient, and prescription registers. Household disposable income and family type (2016) were examined as moderators. Cox regression models estimated the effect of precarious employment on diagnosed mental disorders, with moderation assessed using two-way interaction terms.

**Results:**

The risk of diagnosed mental disorders was higher among those in PE (HR 1.21, 95% CI 1.18–1.23) compared with those in standard employment, across household income levels and family types. High household income was generally protective, but the interaction between PE and high income indicated that this protective effect was reduced for individuals in PE, both among men (interaction HR 1.22, 95% CI 1.04–1.43) and women (interaction HR 1.25, 95% CI 1.13–1.38). Among women, family types other than “couple without children” amplified the effects of PE on diagnosed mental disorders. The interaction for single mothers (HR 1.27, 95% CI 1.14–1.42) showed that the combined effect of PE and single motherhood exceeded the sum of their separate effects.

**Conclusion:**

Higher household income does not fully buffer the negative impact of PE on diagnosed mental disorders, and the negative impact of PE appears stronger for women, especially mothers. The findings are most generalisable to contexts with similar welfare regimes and gender norms.

**Supplementary Information:**

The online version contains supplementary material available at 10.1186/s12889-026-26259-x.

## Background

The Swedish labour market is increasingly characterized by precarious employment (PE), a multidimensional phenomenon characterized by employment instability, income inadequacy, and lack of rights and protection [1]. PE can be understood in contrast with standard employment (SE), traditionally characterized by stable, often full-time employment with sustainable wages and access to rights and benefits. Employment is an important determinant of health and health inequities [2] and while a growing body of research links PE to mental disorders [3, 4], the mechanisms of action and the differential impact across population subgroups are not fully understood. Meanwhile, mental disorders is a growing cause for concern given tremendous individual and societal costs worldwide [5]. Since both PE and mental disorders are increasingly prevalent in Sweden [6, 7], examining them in conjunction is important.

### The knowledge gap

Despite growing interest in examining the links between PE and mental health, previous occupational health research has rarely considered the multidimensional nature of PE, leaving a gap in understanding the mechanisms and contextual factors involved. Theoretical models suggest pathways through material or social deprivation and psychosocial stress [8] and empirical evidence, primarily from qualitative and cross-sectional research, supports these models [8, 9]. However, while there is theoretical consensus that several contextual factors may modify mental health outcomes associated with PE [10], empirical evidence is lacking. This study addresses this gap by applying a multidimensional operationalization of PE to comprehensive longitudinal register data and by examining how contextual factors at the household level shape the association between PE and diagnosed mental disorders.

### The household context

One key set of contextual factors relates to the household [4], where stressors and resources – such as household income – may influence health outcomes differently for workers in PE compared to workers in SE. Most likely, the relative importance of household resources depends on the welfare state context, since their buffering potential may be most pronounced in countries with weak social support systems [10]. Additionally, social norms and cultural expectations can influence the relative perceived importance of household resources for workers in PE [9]. In this sense, Sweden represents a special case with its relatively generous welfare state, strong dual earner norms, and advances in gender equity [11] – conditions that may shape the buffering potential of household resources for workers in PE. Although previous evidence from other countries suggests that household resources could mitigate adverse health outcomes for workers in PE [12, 13], it is unknown to what extent this holds true in the Swedish context.

Family composition is another key contextual factor that may shape the relationship between PE and mental health, but research on how different family types influence this association remains scarce. Single status is associated with increased mental health risks in the general population, often explained by the lack of social, emotional and financial support a partner might provide [14, 15]. For workers in PE this lack of support could be particularly detrimental. At the same time, some aspects of singlehood—such as greater flexibility and fewer family-related financial obligations—may alleviate stress for workers in PE. Among couples, research indicates that those with lower socioeconomic status experience higher levels of family conflict [16], suggesting that PE may add strain to couple relationships. PE has been conceptualized as a major family stressor [17] with financial strain and work schedule insecurity exacerbating tensions in close relationships and increasing Work-Family Conflict (WFC) [8, 18]. Gender norms around financial contributions and domestic labour may further intensify these dynamics [19]. Moreover, PE has been found to intensify parental stress [8]. For parents, the impact of PE on mental health is likely to depend on multiple factors, including external support, resources, and gender dynamics. Parents in PE may face both economic strain and time pressures, which can compound stress within the family. In contrast, couples without children may experience fewer family-related demands, which could partly explain differences in mental health outcomes between family types.

Overall, the influence of family composition on the relationship between PE and mental health is likely to be complex, reflecting a combination of material, social, and gendered mechanisms. Examining household income and family composition as moderators provides insights not only into these mechanisms but also into where policy efforts to reduce health inequalities might be most effectively targeted. Taken together, these considerations highlight the importance of examining the household context as a potential effect modifier of the PE–mental health relationship.

### The gender perspective

Incorporating a gender perspective is crucial when examining household-level stressors and resources, which are often unequally distributed between men and women [11, 19]. While sex refers to biological characteristics, observed disparities between men and women largely reflect gendered social and cultural inequalities that arise from roles and positions assigned on the basis of sex [20, 21]. These inequalities are expressed through the gendered division of household labour, care responsibilities, and labour market segmentation, which together increase women’s likelihood of being in PE [11]. The hypothesis that men and women derive social value from different domains, with caregiving central to women’s roles and breadwinning to men’s [22], is sometimes used to explain gender differences in mental health impacts of PE [4]. Despite a strong justification to study gendered patterns in the mental health impacts of PE, recent reviews on PE and mental health [4] conclude that gender has been largely ignored in previous research. Thus, our understanding of how gender and household-related factors influence the mental health impacts of PE remains limited.

### Study aim

Research is needed to clarify how contextual factors can modify the harmful effects of PE. While household-related factors may either buffer or exacerbate the negative impact of PE on mental health, this aspect has not yet been studied in the Swedish context. The aim of this study was to investigate the moderating role of household disposable income and family type on the association between PE and diagnosed mental disorders, with consideration for differences between women and men.

## Materials and methods

This longitudinal study used register data from the Swedish Work, Illness, and Labour-market Participation cohort (SWIP). The SWIP cohort contains data from multiple linked registers and includes all individuals aged between 16 and 64 years who were registered in Sweden in the year 2005, approximately 5.4 million individuals. Details about the SWIP cohort are available elsewhere [23].

This study used a subsample of individuals employed in Sweden during the baseline year 2016, who could be followed up for first incidence of diagnosed mental disorders between 2017 and 2019, until death, emigration, or until end of follow-up (31 December 2019). We focused on individuals in employment to examine how differences in employment quality relate to mental health, and therefore excluded unemployed individuals, as unemployment represents a qualitatively distinct state with different mechanisms. Individuals were excluded for the following reasons: older than 65 years, retired or in early retirement, unemployed, on sick leave, inactive in the labour market, student, migrated or died in 2016, or had received a mental disorder diagnosis in 2015 or 2016. A two-year washout period (2015–2016) was applied to reduce the risk of reverse causation and avoid misclassifying ongoing cases as incident events during follow-up (a flow chart of the sample selection is included in Additional file 1). Additionally, individuals who were self-employed in 2016 were excluded since the scoring method used to measure exposure is unsuited to distinguish precarity among this heterogenous group [24]. Finally, given that it is relatively uncommon for adults (> 27 years old) to live with their parent(s) in Sweden, individuals registered at their parent(s) address in 2016 (*n* = 57076) were also excluded to avoid misclassification. The final analytical sample consisted of 2 509 229 individuals.

### Exposure

The study population was grouped into one of three employment exposure categories: precarious (PE), substandard (SSE) or standard (SE), according to the Swedish Register-based Operationalization of Precarious Employment (SWE-ROPE 2.0) [25], using the summative scale approach [23]. The SWE-ROPE operationalization covers three key dimensions of PE: employment insecurity, income inadequacy, and lack of rights and protection [1], using five specific items available in register data: (i) contractual relationship insecurity, (ii) contractual temporariness, (iii) multiple jobs/sectors, (iv) individual income, and (v) likelihood of coverage by collective bargaining agreement. The items receive a score ranging from + 2 to −2, allowing a total score ranging from + 2 to −9 [23]. Participants were classified as being in PE (score < − 3), SSE (score − 3 to − 1), and SE (score 0 to + 2). The SSE group was created to clearly differentiate between PE and SE. The three exposure categories used the same cut-off points as applied in previous studies [26, 27]. The precarious score was calculated using information from employed working population only. Thus, individuals who were students, self-employed, or had no registered income were excluded from the calculation.

It is important to note that while the SWE-ROPE index includes an individual-level income adequacy component, this reflects adequacy of personal earnings relative to subsistence thresholds rather than household resources. This distinction means that the exposure measure captures the precariousness of an individual’s own labour market position, whereas household disposable income, introduced below as an effect modifier, reflects the broader financial situation of the household. Nevertheless, we acknowledge some conceptual overlap between these dimensions and have addressed this empirically in sensitivity analyses and conceptually in the Discussion.

### Outcome

The outcome of interest was first incidence of diagnosed mental disorders during the follow-up period (2017–2019) and was retrieved from nationwide registers of inpatient and specialized outpatient care along with the prescribed drug register. Diagnosed mental disorders was defined as diagnosed (ICD-10 codes in brackets) depressive disorder (F32-F33), anxiety (F41), stress reaction disorders (F43), substance use disorder (F10-F16, F18-F19), self-harm (E95, E98, X6-X7, X80-X84), eating disorder (F50), events of undetermined intent (Y10-Y34), or being prescribed antidepressant or anxiolytic medication (ATC codes N06A or N05B). Both main and contributory diagnoses were included.

### Effect modifiers

To assess the potential moderating effect of the household context, we also gathered data on *household disposable income* (quartiles) and *family type* from the Swedish Longitudinal Integrated Database for Health Insurance and Labour Market Studies (LISA). Regarding *family type*, study participants were categorized as (a) couple without children, (b) couple with children, (c) single, and (d) single parent. As household income is closely related to individual earnings, it may act both as a mediator and as a moderator of the relationship between precarious employment and health. Our aim in using household disposable income as an effect modifier was to examine whether material resources at the household level buffer the risks of precarious employment. At the same time, we recognize the dual role of income and therefore interpret these results with caution.

### Covariates

Covariate selection was informed by a Directed Acyclic Graph (DAG) based on prior literature, to ensure appropriate adjustment for confounding. Four variables were retrieved from the LISA register: *sex* (assigned at birth: male/female); *age*; *level of education* (primary: ≤9 years, secondary: 12 years, higher: ≥13years); and *country of birth* (Sweden, EU, non-EU).

### Statistical analysis

First, the distribution of all covariates was calculated according to employment category (PE, SSE, or SE). Second, Cox proportional hazards regression models were fitted to estimate the association between employment category and the risk of first incidence of diagnosed mental disorders during follow-up, with SE as the reference category. Person-time was calculated from 1 January 2017 until emigration, death, first incidence of diagnosed mental disorders, or end of follow-up (31 December 2019), whichever came first. Hazard Ratios (HR) and 95% Confidence Intervals (95% CI) were estimated.

Third, the moderating effects of household disposable income (quartiles) and family type were examined on the multiplicative scale by adding two-way interaction terms to the main effects models. The reference category for household income was SE with the lowest income quartile, and for family type it was SE couple without children. Because interaction models were used, the main effects correspond to these reference groups, and stratum-specific effects were derived by combining main and interaction terms. This approach captures how the association between employment category and diagnosed mental disorders varies across different household contexts.

Analyses were conducted on the full analytical sample as well as separately by sex, in light of previous research indicating that precarious employment is more prevalent among women and that its effects may differ between men and women [28]. Data management and statistical analyses were conducted using STATA v. 17.

### Sensitivity analyses

We conducted two sets of sensitivity analyses. First, we replicated the interaction analyses using a variant of the SWE-ROPE 2.0 index that excluded the individual income adequacy component to assess potential overlap between exposure and moderator variables. Results are presented in Additional file 2, Supplementary Table 1. Second, we reran the moderation analyses using separate models for each interaction term: employment category × household disposable income and employment category × family type. These results are shown in Additional file 2, Supplementary Tables 2–3.

## Results

Table 1 presents the characteristics of the study population. At baseline, 8% of participants were classified as being in PE, 30% in SSE, and 62% in SE. The distribution by sex varied: SE included 43% women, SSE 58%, and PE had a nearly equal distribution (51% women). Compared to those in SE, individuals in PE were more likely to have been born outside of Sweden (20% vs. 10%), to have primary education only (15% vs. 6%), to belong to the youngest age quartile (37% vs. 16%), and to fall within the lowest household income quartile (69% vs. 13%). They also had a higher incidence of diagnosed mental disorders during follow-up (8% vs. 5%).

**Table 1:** Characteristics of the study sample (*n* = 2 509 229)

	**Precarious Employment (PE)**		**Substandard Employment (SSE)**		**Standard Employment (SE)**	
	n	%	n	%	n	%
Sex						
Male	92 637	49	314 555	42	899 075	57
Female	96 370	51	436 386	58	670 206	43
Country of birth						
Sweden	150 460	80	649 600	86	1 412 612	90
EU	21 411	11	51 914	7	76 370	5
Non-EU	17 136	9	49 427	7	80 299	5
Level of education 2016						
Primary	28 991	15	77 212	10	100 087	6
Secondary	44 784	24	179 391	24	338 336	22
Higher	115 232	61	494 338	66	1 130 858	72
Age 2016 Median (IQR)	41 (19)		42 (19)		47 (15)	
27–35	69 603	37	241 669	32	246 724	16
36–45	47 402	25	204 475	27	448 631	28
46–55	44 821	24	187 614	25	534 783	34
56–65	27 181	14	117 183	16	339 143	22
Household disp. Inc. 2016 Median (IQR)*	1844 (1083)		2461 (1048)		3157 (1376)	
Quartile 1 (lowest income)	130 641	69	297 395	40	203 551	13
Quartile 2	28 896	15	212 252	28	395 257	25
Quartile 3	17 090	9	150 345	20	464 459	30
Quartile 4 (highest income)	12 380	7	90 949	12	506 014	32
Family type 2016						
Couple without children	18 400	10	94 014	12	253 593	16
Couple with children	75 369	40	366 314	49	788 660	50
Single	77 699	41	230 455	31	414 714	27
Single parent	17 539	9	60 158	8	112 314	7
Mental ill-health during follow-up**	14 233	8	57 847	8	83 042	5

*Equivalized per consumption unit. *IQR* Inter quartile range in hundreds of Swedish Krona

**First incidence of diagnosed mental disorder (2017–2019) or treatment with psychotropic drugs (2017–2019)

Table 2 presents the main effects from the fully adjusted Cox regression models, without interaction terms. In these models, the reference categories are standard employment for employment category, lowest household income quartile for income, and couple without children for family type. Both PE and SSE were associated with a higher risk of diagnosed mental disorders compared to SE, and women had a consistently higher risk of diagnosed mental disorders than men. Higher household income, higher education, older age, and being born in Sweden were all associated with lower risk. Regarding family type, compared to couples without children, being in a couple with children was associated with lower risk, whereas both single and single parent family types were associated with increased risk in both sexes. All reported differences were statistically significant.

**Table 2:** Precarious Employment (2016) and diagnosed mental disorder (2017-2019)*, stratified by sex (*n*=2 509 229), fully adjusted model

	Men		Women		Total	
	HR	CI95%	HR	CI95%	HR	CI
Standard employment (ref)	1	.	1	.	1	.
Substandard employment	1.17	1.15–1.19	1.22	1.20–1.24	1.21	1.20–1.22
Precarious employment	1.20	1.16–1.24	1.19	1.16–1.22	1.21	1.18–1.23
Household disp. inc. 2016						
Quartile 1 (lowest) (ref)	1	.	1	.	1	.
Quartile 2	0.91	0.89–0.94	0.92	0.91–0.94	0.92	0.91–0.94
Quartile 3	0.83	0.81–0.85	0.86	0.84–0.87	0.85	0.84–0.87
Quartile 4 (highest)	0.74	0.72–0.77	0.78	0.77–0.80	0.78	0.76–0.79
Family type 2016						
Couple without children (ref)	1	.	1	.	1	.
Couple with children	0.87	0.84–0.90	0.94	0.92–0.97	0.93	0.91–0.95
Single	1.18	1.14–1.21	1.09	1.06–1.12	1.14	1.11–1.16
Single parent	1.27	1.22–1.33	1.28	1.24–1.32	1.29	1.25–1.32
Age						
27–35 (ref)	1	.	1	.	1	.
36–45	0.91	0.89–0.94	0.89	0.87–0.90	0.90	0.88–0.91
46–55	0.83	0.81–0.85	0.74	0.72–0.75	0.77	0.76–0.78
56–65	0.77	0.75–0.80	0.62	0.60–0.63	0.67	0.66–0.68
Country of birth						
Sweden (ref)	1	.	1	.	1	.
EU	1.23	1.19–1.27	1.10	1.07–1.13	1.14	1.12–1.16
Non-EU	1.26	1.22–1.30	1.29	1.25–1.32	1.27	1.25–1.30
Level of education						
Primary (ref)	1	.	1	.	1	.
Secondary	0.88	0.86–0.91	0.95	0.92–0.98	0.91	0.89–0.93
Higher	0.79	0.77–0.82	0.88	0.86–0.91	0.83	0.82–0.85
Sex						
Male (ref)					1	.
Female					1.72	1.70–1.74

*First incidence of diagnosed mental disorder (2017–2019) or treatment with psychotropic drugs (2017–2019), adjusted for all covariates

### Effect modification

Table 3 shows the fully adjusted model including both interaction terms: Employment category*Household disposable income, and Employment category*Family type. Because Table 3 presents results from interaction models, the main effects correspond to the reference categories (standard employment, lowest household income quartile, and couples without children). Stratum-specific effects are obtained by combining these main effects with the relevant interaction terms. For clarity, stratum-specific hazard ratios are also plotted in Additional file 3.

**Table 3:** Precarious employment and diagnosed mental disorder*. Interaction with household income and family type (*n* = 2 509 229), fully adjusted model

	Men		Women		Total	
	HR	CI95%	HR	CI95%	HR	CI95%
Standard employment (ref)	1	.	1	.	1	.
Substandard employment	1.17	1.08–1.26	1.15	1.09–1.22	1.15	1.10–1.20
Precarious employment	1.09	0.97–1.24	0.95	0.86–1.05	1.01	0.93–1.09
Household disp. inc. 2016						
Quartile 1 (lowest) (ref)	1	.	1	.	1	.
Quartile 2	0.92	0.89–0.95	0.93	0.90–0.96	0.93	0.91–0.95
Quartile 3	0.82	0.79–0.85	0.83	0.81–0.86	0.84	0.82–0.86
Quartile 4 (highest)	0.73	0.70–0.75	0.74	0.72–0.77	0.75	0.73–0.76
Family type 2016						
Couple without children (ref)	1	.	1	.	1	.
Couple with children	0.86	0.82–0.89	0.91	0.88–0.94	0.89	0.86–0.91
Single	1.19	1.14–1.23	1.09	1.05–1.13	1.14	1.11–1.17
Single parent	1.25	1.18–1.32	1.22	1.17–1.27	1.23	1.19–1.27
Interaction effects						
Employment category * Household disp. inc.						
Standard*Q1 (lowest) (ref)	1	.	1	.	1	.
Substandard*Q2	0.96	0.91–1.01	0.96	0.92–1.00.92.00	0.96	0.93–0.99
Substandard*Q3	1.00	0.95–1.06	1.04	0.99–1.08	1.02	0.98–1.05
Substandard*Q4 (highest)	1.09	1.01–1.17	1.11	1.06–1.17	1.10	1.06–1.15
Precarious*Q2	1.03	0.94–1.12	1.08	1.01–1.16	1.05	1.00–1.11.00.11
Precarious*Q3	1.10	0.98–1.24	1.11	1.01–1.21	1.09	1.02–1.17
Precarious*Q4 (highest)	1.22	1.04–1.43	1.25	1.13–1.38	1.22	1.12–1.33
Employment category*Family type						
Standard*couple without children (ref)	1	.	1	.	1	.
Substandard*couple with children	1.03	0.96–1.10	1.06	1.01–1.11	1.09	1.04–1.13
Substandard*single	0.97	0.90–1.04	0.99	0.94–1.05	0.99	0.95–1.03
Substandard*single parent	1.03	0.93–1.15	1.06	1.00–1.13.00.13	1.07	1.01–1.12
Precarious*couple with children	1.12	0.99–1.27	1.23	1.12–1.35	1.21	1.12–1.31
Precarious*single	1.04	0.92–1.18	1.12	1.01–1.24	1.12	1.03–1.21
Precarious*single parent	1.09	0.91–1.30	1.27	1.14–1.42	1.21	1.10–1.32

*First incidence of diagnosed mental disorder (2017–2019) or treatment with psychotropic drugs (2017–2019), adjusted for all covariates (age, country of birth, level of education, sex) and interaction terms

### Household disposable income

While a higher household income was associated with a lower risk of diagnosed mental disorders (Table 2), a significant interaction between PE and the highest income quartile (HR = 1.22, 95% CI 1.12–1.33) shows that the hazard ratio for PE differed across income strata, with a higher HR observed among individuals in the highest income quartile compared to the reference group (lowest income quartile, SE).

### Family composition

The elevated risk associated with PE (vs. SE) was observed across all family types. The interaction between PE and “couple with children” (HR = 1.21, 95% CI 1.12–1.31) indicates that the HR for PE differed between couples with children and the reference group (SE, couples without children).

Among women in PE, all other family types showed significant interaction effects compared to the reference group, whereas no statistically significant interaction effects by family type were observed among men. Across all employment types, single parents had the highest risk, with the largest interaction term observed among single mothers in PE (HR = 1.27, 95% CI 1.14–1.42).

## Discussion

### Main findings

This study examined whether household disposable income and family type moderate the relationship between precarious employment (PE) and diagnosed mental disorders. Results show that PE, compared to standard employment (SE), is linked to a higher risk of diagnosed mental disorders for both men and women across various income levels and family types. While higher household income generally buffers against diagnosed mental disorders, it appears less protective for those in PE (compared to SE), particularly women. Individuals in couple relationships have a lower risk of diagnosed mental disorders compared to single individuals or single parents, with single mothers facing the highest risk. Gender differences within the PE group show that higher household income is even less protective for women, and synergistic interaction effects from family types are only observed in women, suggesting PE is especially harmful to women.

### Household disposable income

We found a low correlation between individual and household incomes in our sample, indicating that PE workers can have high household income. Results show consistent elevated risk of mental disorders for PE workers, especially women, irrespective of household income. Thus, higher household income does not fully mitigate the adverse mental health effects associated with PE. While results show a general buffering effect of higher household income on diagnosed mental disorders, in line with existing theory and evidence [29], the interaction patterns likely reflect both mediation and moderation mechanisms. For lower-income households, the material pathway may explain a larger part of the association between PE and mental health, whereas among higher-income households, other dimensions of precariousness (e.g., employment insecurity, lack of rights) may play a more prominent role. Interpreting the findings through this dual lens provides a nuanced understanding of the observed interaction.

The findings provide both conceptual and methodological insights. First, the persistence of elevated mental health risks among individuals in PE, even at higher levels of household income, indicates that the adverse effects are not driven solely by limited material resources. This highlights the importance of a multidimensional approach to understanding PE and its impact on mental health. Our findings support previous research on PE underscoring its multidimensional nature, where several aspects of precarity, beyond low individual-level income, are used to explain the influence of PE on workers’ mental health. These additional dimensions include employment instability and lack of rights and protection [1], which may all lead to subjective insecurity and stress and, in turn, impact mental health.

Second, even where limited material resources are the main issue, income volatility or unpredictability may not be captured in the accumulated yearly amount measured retrospectively in register data. For example, qualitative research has found that workers in PE experience work intensification due to schedule and income insecurity, including working long hours, de-prioritizing rest and social activities, and accepting bad conditions to secure future income [9]. Similarly, Irvine and Rose [8] find that several “core experiences” of PE lead to behavioral responses including overwork, presenteeism and acceptance of hazardous conditions [8]. These actions may keep the yearly income at an acceptable level while being detrimental for mental health in the day-to-day, again highlighting why PE-related challenges are not adequately captured by yearly income alone.

At the same time, it is important to acknowledge that income may act both as a mediator and as a moderator of the relationship between PE and mental health. On the one hand, low earnings are part of the multidimensional SWE-ROPE index and reflect individual income adequacy; on the other, household disposable income represents broader household resources and was introduced as a potential effect modifier. While the modest correlation between these measures suggests that they capture related but distinct constructs, some conceptual overlap remains. Importantly, preliminary sensitivity analyses in which the income component was excluded from the PE measure yielded similar results, indicating that the observed effect modification is not driven by scale construction but likely reflects a substantive interaction between employment precarity and household resources. Future studies using causal mediation analyses would be valuable to disentangle the extent to which household income mediates, moderates, or jointly influences the association between PE and mental health. It is also important to acknowledge that the modest correlation between individual and household income may differ across subgroups. Among individuals in the lowest household income quartile and among single-parent families, individual earnings likely contribute more directly to total household resources, meaning that adjusting for household income may ‘wash out’ part of the material pathway from precarious employment to mental health. This may help explain why the estimated association between PE and mental disorders was weaker in these groups. Conversely, the elevated risk observed among PE workers in higher-income households may reflect psychosocial mechanisms such as status incongruence or unmet role expectations, which merit investigation in future studies.

### Family composition

The lower risk of diagnosed mental disorders for individuals in couple relationships, observed in the main effects model (Table 2), suggests that living with a partner may buffer some of the stressors associated with work and life more broadly. This may reflect the protective influence of shared financial and social resources, which appears to outweigh potential strains related to gendered divisions of labour or work–family conflict that might otherwise increase stress within couples. The findings are in line with research on the general population showing benefits of social relations [30] and detrimental effects of isolation [31] and are therefore not surprising. However, there may also be some selection effect as PE can be detrimental to family formation and family cohesion [17, 19], whereby the most precarious individuals are also likely to be found in the single and single parent categories.

A second noteworthy pattern pertains to the increased risk of diagnosed mental disorders for parents in our sample. Results indicate that parents in PE, both in couple relationships and as single parents, face an added vulnerability compared to their counterparts in SE. This pattern could be explained by socio-relational stress within the family [8, 17] and WFC [18, 19]. Research on PE and mental health outcomes has connected socio-relational stress to impaired parental mental health [8] and also found spill-over effects on children’s mental health [26]. It is argued that several core dimensions of PE (e.g., financial instability and temporal uncertainty) lead to socio-relational stress such as conflicts and tension within the family, which, in turn, impact mental health via increased levels of WFC [8]. In line with this, the “employment precarity family stress model”, underscores the role of economic pressure for the increased socio-relational strain [17].

### Gender differences

Results revealed notable gender differences, pointing to a particular vulnerability for women in PE, especially mothers. For men in PE, results showed that although the predicted risk of diagnosed mental disorders varied across family types, being highest among single fathers, there were no significant interaction effects with PE. This can be interpreted as being a single father implies an added stress in general, but this stress is not greater for single fathers in PE compared to single fathers in SE. Among women, however, especially family types that included children had a synergistic interaction effect on the association between PE and diagnosed mental disorders (i.e., compounded the adverse effects) (Table 3). The interesting finding that family type compounded mental health risk only for women suggests that gender norms may play a role, in line with existing social role theory and empirical evidence [19, 22].

Although Sweden is a country with strong dual-earner norms that scores high on gender equality [11, 32], the negative health implications of WFC remain. In fact, Hagqvist et al. [32] found that even when parents living in countries advanced in gender equality report lower levels of WFC, the negative impact on wellbeing is stronger compared to countries with more traditional gender norms. The authors note that “parallel ideals seem to exist” [32] in Nordic countries, where there are strong expectations of women to participate in breadwinning activities, but also persisting socio-cultural norms that see women as the main caregivers. The negative implications of WFC may thus be particularly strong in a country like Sweden due to unmet expectations to successfully fulfill both roles. These gendered vulnerabilities may be partly explained by other psychosocial mechanisms such as the persistent unequal distribution of caregiving responsibilities, limited social support, and gender norms in the labor market, which together intensify the burden of precarious work for women, particularly single mothers [33].

Finally, while not a primary focus of this study, it is worth noting that the associations observed for the SSE group were of similar magnitude to those for PE in some strata. Although the SWE-ROPE was designed to differentiate levels of employment quality, the absence of a strict gradient in the mental health outcomes suggests that even moderately disadvantaged employment conditions can confer substantial mental health risks. This pattern warrants further investigation in future research, both to better understand the specific aspects of SSE contributing to risk and to clarify whether these similarities reflect conceptual features of the multidimensional instrument or unmeasured psychosocial stressors not captured by household income.

### Strengths and limitations

Working with register data has tremendous advantages, including large sample size and low attrition during follow-up. Register data also eliminates the issue of recall bias and self-report bias, as both exposure and outcome are extracted from national registers. Another important strength lies in the use of the SWE-ROPE 2.0 measure to operationalize PE. This measure shares conceptual similarities with other multidimensional instruments such as the EPRES scale, capturing key dimensions including income inadequacy, employment insecurity, and lack of rights and protection. Unlike survey-based instruments, SWE-ROPE was specifically developed for Swedish register data, allowing for population-wide coverage and longitudinal analyses without self-reports. While this limits direct comparability with studies from other contexts, it strengthens measurement validity and enables a robust analysis of PE in the Swedish context.

There are, however, limitations, for example regarding the way in which family composition can be operationalized. In Swedish register data, a family can be identified based on marital status and/or parent-child relationship. We were therefore unable to identify cohabiting couples without children and other family constellations where individuals may or may not be registered at the same address but still potentially share resources with each other. Similarly, we assume that individuals in the same household share resources and that a higher household disposable income will benefit the individual, which may not always be the case. Furthermore, several sources of income fall outside the register measurement of household disposable income, for example financial support from parents to children (who no longer live with the parents), gifts between households, remittances and so called “black” or undeclared income. Additionally, Swedish register data does not include information on WFC or division of labour in the home.

Another aspect that warrants consideration is the role of income in our study design. While individual-level income adequacy forms part of the SWE-ROPE index, household disposable income was also used as a potential moderator. This introduces some conceptual overlap and highlights the possibility that household income may function simultaneously as a mediator and a moderator of the PE–mental health relationship. Although we did not conduct formal mediation analyses, we addressed this issue conceptually and through sensitivity analyses, and our findings on income moderation should be interpreted in light of this dual role.

It should also be noted that unemployed individuals were excluded from the analytic sample. This choice was motivated by our focus on employment precarity among those currently employed, as unemployment represents a qualitatively distinct labor market state. However, we acknowledge that precarious employment may increase the risk of job loss, and that excluding unemployment spells may therefore underestimate the total health burden associated with precarious employment. Transitions between precarious employment and unemployment should be addressed in future research.

A further limitation relates to the use of administrative health registers to identify cases of mental disorders. While these data sources have the advantage of complete national coverage and avoid self-report bias, they may underrepresent milder or moderate cases that do not reach specialized care or prescription records. Moreover, health care utilization is known to vary across sociodemographic groups, with disadvantaged populations in particular facing barriers to accessing mental health services. As a result, our outcome measure may underestimate the true prevalence of mental disorders, and differential detection across social groups could lead to conservative estimates of inequalities.

A methodological point worth noting is that interaction analyses were conducted on the multiplicative scale only. Future studies could extend this work by incorporating additive interaction measures (e.g., RERI, AP), which would allow for quantification of the absolute excess risk attributable to joint exposures and enhance the public health interpretation of the findings.

Another methodological consideration relates to the use of a static household income measure from 2016. While dynamic income trajectories can provide valuable insights, the relatively short follow-up period and the stable pre-pandemic economic context make it unlikely that income fluctuations would have substantially influenced our findings. In fact, focusing on a stable period can be seen as a strength, as it allows for examining the PE–mental health association without the confounding influence of major labour market shocks. Future studies could complement this approach by exploring how income dynamics over longer periods shape these relationships.

Finally, it is important to note that the available information is limited to sex assigned at birth (male/female). Although our analyses are stratified by this variable, the observed differences are interpreted through a gender lens, since social and cultural norms shape health risks along the axis of sex. Future research should incorporate measures of gender identity to move beyond the male–female binary and strengthen the analysis of health inequalities.

### Implications for policy and future research

While no efforts should be spared to restore a fair and complete rights-based employment contract, results point to a need for harm-reduction interventions. Ensuring sufficient income through fair wages and benefits is essential but not enough, and policymakers should aim to address other dimensions associated with PE such as employment and schedule insecurity, especially for women and single mothers who appear most vulnerable. The gendered nature of the findings highlights their policy relevance and points to the need for coordinated labour market and family policies. Measures to reduce PE should be complemented by policies that support caregiving responsibilities and tackle persistent gender inequalities in both paid and unpaid work. This includes ensuring accessible childcare, strengthening social safety nets, and promoting more equitable divisions of care. Aligning labour market and family policies in this way is especially important in contexts like Sweden, where dual-earner norms coexist with enduring gendered expectations around care. Continuous research and monitoring are essential to understand the evolving needs of workers in PE and the effectiveness of implemented policies. Finally, the findings should be interpreted in light of the Swedish context and are most directly generalizable to countries with welfare regimes, dual-earner norms, and gender equity levels similar to Sweden. Patterns may differ in contexts with weaker social protection or more traditional gender norms.

## Conclusions

Precarious employment is consistently associated with a higher risk of diagnosed mental disorders compared to standard employment, though the magnitude of this risk varies by gender, household income level, and family type. While higher household income mitigates some of the risk, its protective effect is less pronounced for individuals in precarious employment—especially women, who remain at elevated risk even at higher income levels. These findings suggest that other dimensions of precariousness—such as instability and lack of rights—may override the protective role of income. Importantly, this study provides longitudinal evidence of gendered vulnerabilities, showing that women in precarious employment, particularly mothers, face a compounded risk of diagnosed mental disorders, highlighting the need for coordinated labour market and family policies. Overall, the results indicate that precarious employment is a structural risk factor for mental health that cannot be fully offset by household resources or social support systems, highlighting the need for policy measures aimed at improving employment conditions and reducing precariousness for all workers.

## Supplementary Information

Additional file 1: Flow chart of sample selection

Additional file 2: Sensitivity analyses

Additional file 3: Stratum specific hazard ratios (plots)

## Data Availability

The data underlying the results of this study is available from Statistics Sweden and the Swedish National Board of Health and Welfare and was used for the current study under license after ethical review. Hence, the data is not publicly available. For questions pertaining to this data, contact the corresponding author of this study.

## Abbreviations

DAG: Directed Acyclic Graph
EPRES: Employment Precariousness Scale
LISA: Swedish Longitudinal Integrated Database for Health Insurance and Labour Market Studies
PE: Precarious Employment
SE: Standard Employment
SSE: Substandard Employment
SWE-ROPE: Swedish Registered-based Operationalization of Precarious Employment
SWIP: Swedish Work, Illness, and Labour-market Participation cohort
WFC: Work-Family Conflict
