# Supplementary material for: Precarious employment and mental health: the moderating role of household income and family type in Sweden
Source: BMC Public Health. 2026 Jan 27;26:349. doi: 10.1186/s12889-026-26259-x (PMC12849647; doi:10.1186/s12889-026-26259-x)
Supplement: Supplementary file 1 — Additional file 1: Flow chart of sample selection [file 12889_2026_26259_MOESM1_ESM.pdf]

Additional file 1. Flowchart of sample selection.

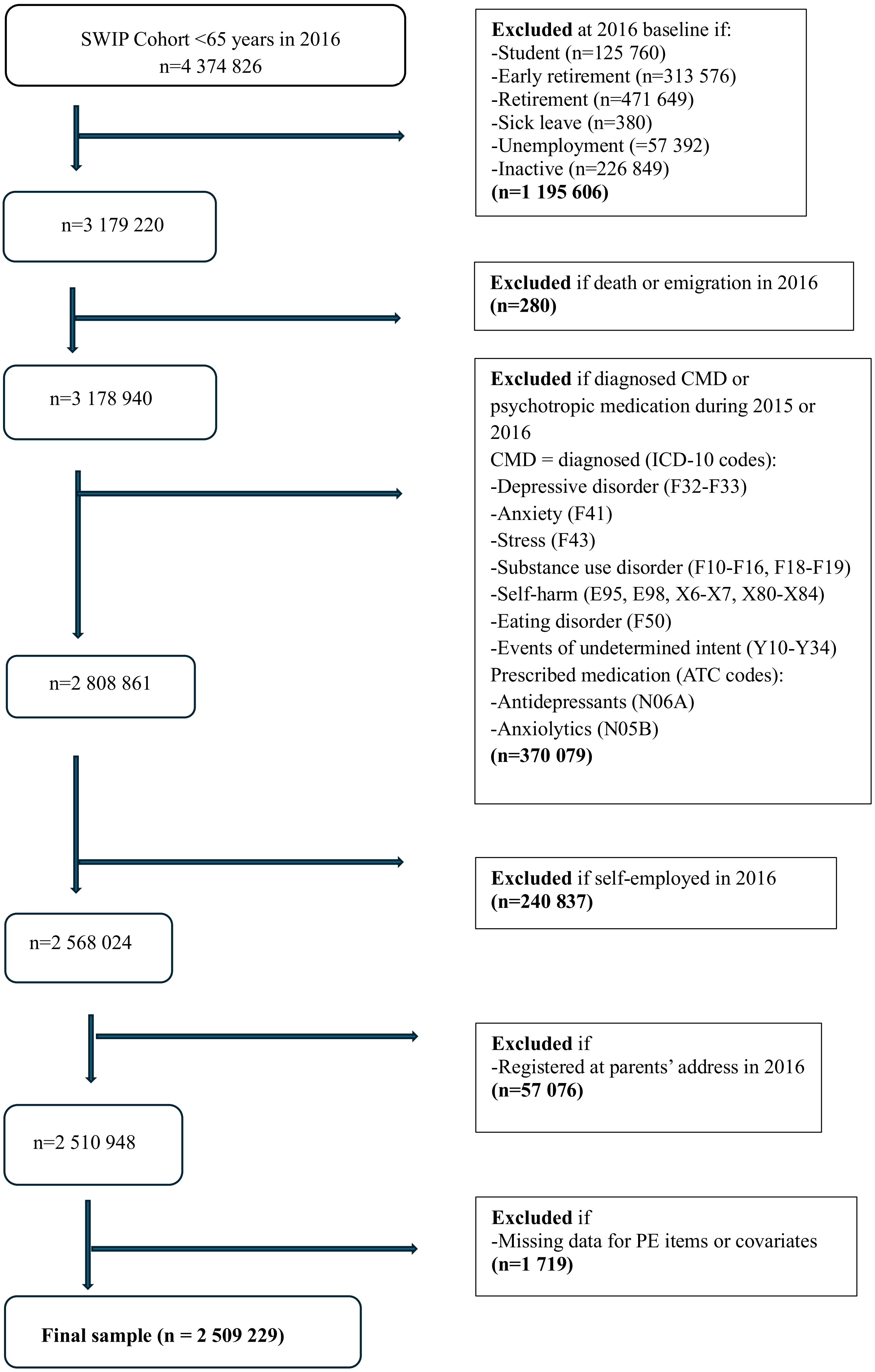

Additional file 1. Flowchart of sample selection.
