# Supplementary material for: Precarious employment and mental health: the moderating role of household income and family type in Sweden
Source: BMC Public Health. 2026 Jan 27;26:349. doi: 10.1186/s12889-026-26259-x (PMC12849647; doi:10.1186/s12889-026-26259-x)
Supplement: Supplementary file 2 — Additional file 2: Sensitivity analyses [file 12889_2026_26259_MOESM2_ESM.docx]

**Additional file 2: Sensitivity analyses**

Supplementary Table 1 shows the fully adjusted interaction model using a variant of the exposure variable where individual income adequacy was excluded from the SWE-ROPE 2.0 PE-index.

| Supplementary Table 1. Precarious Employment (excluding individual income adequacy) and diagnosed mental disorder*. Interaction with household disposable income and family type (*n*=2 509 229). | | | | | | |
| --- | --- | --- | --- | --- | --- | --- |
|  | **Men** | | **Women** | | **Total** | |
|  | HR | CI95% | HR | CI95% | HR | CI95% |
| Standard employment (ref) | 1 | . | 1 | . | 1 | . |
| Substandard employment | 0.99 | 0.92-1.07 | 0.97 | 0.91-1.03 | 0.98 | 0.94-1.03 |
| Precarious employment | 1.01 | 0.91-1.13 | 0.88 | 0.79-0.97 | 0.95 | 0.88-1.02 |
| Household disp. inc. 2016 |  |  |  |  |  |  |
| Quartile 1 (lowest) (ref) | 1 | . | 1 | . | 1 | . |
| Quartile 2 | 0.87 | 0.84-0.89 | 0.86 | 0.84-0.88 | 0.86 | 0.85-0.88 |
| Quartile 3 | 0.76 | 0.74-0.79 | 0.78 | 0.76-0.79 | 0.78 | 0.76-0.79 |
| Quartile 4 (highest) | 0.67 | 0.63-0.68 | 0.68 | 0.66-0.70 | 0.68 | 0.67-0.70 |
| Family type 2016 |  |  |  |  |  |  |
| Couple without children (ref) | 1 | . | 1 | . | 1 | . |
| Couple with children | 0.83 | 0.80-0.87 | 0.89 | 0.87-0.92 | 0.88 | 0.86-0.90 |
| Single | 1.16 | 1.12-1.21 | 1.04 | 1.00-1.07 | 1.10 | 1.07-1.13 |
| Single parent | 1.18 | 1.12-1.25 | 1.17 | 1.13-1.21 | 1.18 | 1.15-1.22 |
| Interaction effects |  |  |  |  |  |  |
| Employment category * Household disp. inc. |  |  |  |  |  |  |
| Standard*Q1 (lowest) (ref) | 1 | . | 1 | . | 1 | . |
| Substandard*Q2 | 1.00 | 0.95-1.06 | 1.05 | 1.01-1.09 | 1.03 | 0.10-1.06 |
| Substandard*Q3 | 1.05 | 1.00-1.11 | 1.04 | 1.00-1.09 | 1.04 | 1.01-1.08 |
| Substandard*Q4 (highest) | 1.11 | 1.04-1.18 | 1.09 | 1.04-1.15 | 1.09 | 1.05-1.14 |
| Precarious*Q2 | 1.07 | 0.99-1.15 | 1.15 | 1.08-1.23 | 1.11 | 1.06-1.16 |
| Precarious*Q3 | 1.13 | 1.04-1.22 | 1.21 | 1.13-1.30 | 1.16 | 1.10-1.22 |
| Precarious*Q4 (highest) | 1.20 | 1.10-1.31 | 1.27 | 1.17-1.37 | 1.23 | 1.16-1.30 |
| Employment category*Family type |  |  |  |  |  |  |
| Standard*couple without children (ref) | 1 | . | 1 | . | 1 | . |
| Substandard*couple with children | 1.04 | 0.97-1.12 | 1.12 | 1.06-1.18 | 1.08 | 1.03-1.13 |
| Substandard*single | 1.04 | 0.97-1.12 | 1.06 | 1.00-1.13 | 1.05 | 1.01-1.10 |
| Substandard*single parent | 1.10 | 0.99-1.22 | 1.12 | 1.05-1.20 | 1.10 | 1.04-1.17 |
| Precarious*couple with children | 1.07 | 0.96-1.19 | 1.17 | 1.06-1.29 | 1.10 | 1.03-1.19 |
| Precarious*single | 1.04 | 0.94-1.16 | 1.13 | 1.03-1.25 | 1.09 | 1.02-1.18 |
| Precarious*single parent | 1.15 | 0.99-1.34 | 1.23 | 1.10-1.38 | 1.16 | 1.07-1.27 |
| *First incidence of diagnosed mental disorder (2017-2019) or treatment with psychotropic drugs (2017-2019), adjusted for all covariates (age, country of birth, level of education, sex) and interaction terms. | | | | | | |

Supplementary Tables 2-3 show the interaction models estimated separately (using original exposure variable including individual income adequacy).

| Supplementary Table 2. Precarious Employment (including individual income adequacy) and diagnosed mental disorder*. Interaction with household disposable income (*n*=2 509 229). | | | | | | |
| --- | --- | --- | --- | --- | --- | --- |
|  | **Men** | | **Women** | | **Total** | |
|  | HR | CI95% | HR | CI95% | HR | CI95% |
| Standard employment (ref) | 1 | . | 1 | . | 1 | . |
| Substandard employment | 1.18 | 1.14-1.23 | 1.22 | 1.18-1.25 | 1.22 | 1.19-1.25 |
| Precarious employment | 1.18 | 1.13-1.24 | 1.15 | 1.11-1.20 | 1.19 | 0.15-1.22 |
| Household disp. inc. 2016 |  |  |  |  |  |  |
| Quartile 1 (lowest) (ref) | 1 | . | 1 | . | 1 | . |
| Quartile 2 | 0.93 | 0.90-0.96 | 0.94 | 0.91-0.97 | 0.94 | 0.92-0.96 |
| Quartile 3 | 0.83 | 0.80-0.86 | 0.85 | 0.82-0.87 | 0.85 | 0.83-0.87 |
| Quartile 4 (highest) | 0.74 | 0.71-0.76 | 0.76 | 0.74-0.79 | 0.76 | 0.74-0.78 |
| Family type 2016 |  |  |  |  |  |  |
| Couple without children (ref) | 1 | . | 1 | . | 1 | . |
| Couple with children | 0.87 | 0.84-0.90 | 0.94 | 0.92-0.97 | 0.92 | 0.91-0.94 |
| Single | 1.18 | 1.14-1.22 | 1.09 | 1.06-1.12 | 1.13 | 1.11-1.16 |
| Single parent | 1.27 | 1.21-1.33 | 1.28 | 1.24-1.32 | 1.28 | 1.25-1.32 |
| Interaction effects |  |  |  |  |  |  |
| Employment category * Household disp. inc. |  |  |  |  |  |  |
| Standard*Q1 (lowest) (ref) | 1 | . | 1 | . | 1 | . |
| Substandard*Q2 | 0.94 | 0.90-0.99 | 0.95 | 0.91-0.98 | 0.95 | 0.92-0.97 |
| Substandard*Q3 | 0.98 | 0.93-1.04 | 1.01 | 0.97-1.06 | 0.99 | 0.96-1.03 |
| Substandard*Q4 (highest) | 1.07 | 1.00-1.15 | 1.08 | 1.03-1.14 | 1.07 | 1.03-1.11 |
| Precarious*Q2 | 1.02 | 0.93-1.11 | 1.05 | 0.98-1.13 | 1.04 | 0.98-1.09 |
| Precarious*Q3 | 1.08 | 0.96-1.21 | 1.05 | 0.96-1.15 | 1.05 | 0.98-1.12 |
| Precarious*Q4 (highest) | 1.19 | 1.02-1.40 | 1.18 | 1.07-1.30 | 1.16 | 1.07-1.26 |
| * First incidence of diagnosed mental disorder (2017-2019) or treatment with psychotropic drugs (2017-2019), adjusted for all covariates (age, country of birth, level of education, sex) and the interaction term. | | | | | | |

| Supplementary Table 3. Precarious Employment (including individual income adequacy) and diagnosed mental disorder*. Interaction with family type (*n*=2 509 229). | | | | | | |
| --- | --- | --- | --- | --- | --- | --- |
|  | **Men** | | **Women** | | **Total** | |
|  | HR | CI95% | HR | CI95% | HR | CI95% |
| Standard employment (ref) | 1 | . | 1 | . | 1 | . |
| Substandard employment | 1.19 | 1.12-1.27 | 1.22 | 1.17-1.27 | 1.20 | 1.16-1.24 |
| Precarious employment | 1.17 | 1.04-1.30 | 1.07 | 0.98-1.16 | 1.10 | 1.02-1.17 |
| Household disp. inc. 2016 |  |  |  |  |  |  |
| Quartile 1 (lowest) (ref) | 1 | . | 1 | . | 1 | . |
| Quartile 2 | 0.91 | 0.89-0.93 | 0.92 | 0.90-0.94 | 0.92 | 0.91-0.94 |
| Quartile 3 | 0.83 | 0.81-0.85 | 0.85 | 0.84-0.87 | 0.85 | 0.84-0.86 |
| Quartile 4 (highest) | 0.74 | 0.72-0.76 | 0.80 | 0.76-0.80 | 0.77 | 0.76-0.79 |
| Family type 2016 |  |  |  |  |  |  |
| Couple without children (ref) | 1 | . | 1 | . | 1 | . |
| Couple with children | 0.87 | 0.83-0.90 | 0.92 | 0.89-0.95 | 0.90 | 0.88-0.92 |
| Single | 1.20 | 1.15-1.24 | 1.11 | 1.07-1.15 | 1.15 | 1.12-1.18 |
| Single parent | 1.26 | 1.20-1.34 | 1.26 | 1.21-1.31 | 1.26 | 1.22-1.30 |
| Interaction effects |  |  |  |  |  |  |
| Employment category*Family type |  |  |  |  |  |  |
| Standard*couple without children (ref) | 1 | . | 1 | . | 1 | . |
| Substandard*couple with children | 1.00 | 0.93-1.07 | 1.02 | 0.97-1.06 | 1.04 | 1.01-1.08 |
| Substandard*single | 0.94 | 0.88-1.01 | 0.95 | 0.90-1.00 | 0.95 | 0.92-0.99 |
| Substandard*single parent | 1.01 | 0.91-1.11 | 1.01 | 0.95-1.07 | 1.02 | 0.98-1.07 |
| Precarious*couple with children | 1.07 | 0.95-1.22 | 1.16 | 1.06-1.27 | 1.15 | 1.07-1.24 |
| Precarious*single | 1.00 | 0.89-1.13 | 1.04 | 0.94-1.14 | 1.05 | 0.98-1.13 |
| Precarious*single parent | 1.03 | 0.87-1.23 | 1.16 | 1.05-1.28 | 1.12 | 1.03-1.22 |
| * First incidence of diagnosed mental disorder (2017-2019) or treatment with psychotropic drugs (2017-2019), adjusted for all covariates (age, country of birth, level of education, sex) and the interaction term. | | | | | | |
