# Supplementary material for: Precarious employment and mental health: the moderating role of household income and family type in Sweden
Source: BMC Public Health. 2026 Jan 27;26:349. doi: 10.1186/s12889-026-26259-x (PMC12849647; doi:10.1186/s12889-026-26259-x)
Supplement: Supplementary file 3 — Additional file 3: Stratum specific hazard ratios (plots) [file 12889_2026_26259_MOESM3_ESM.docx]

Additional File 3: Adjusted hazard ratios for the association between employment conditions and diagnosed mental disorders stratified by income level (Figure 1) and stratified by family types (Figure 2).


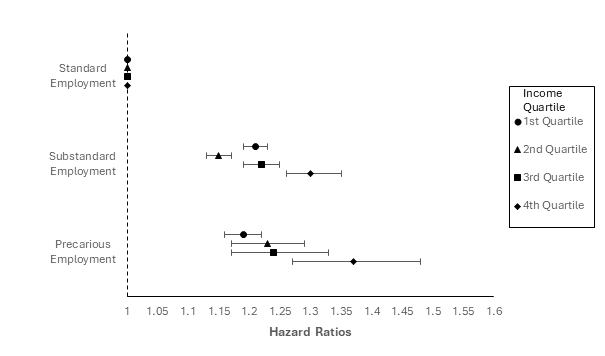


Figure 1: Adjusted hazard ratios for the association between employment conditions and diagnosed mental disorders stratified by income level, Q1 = lowest.


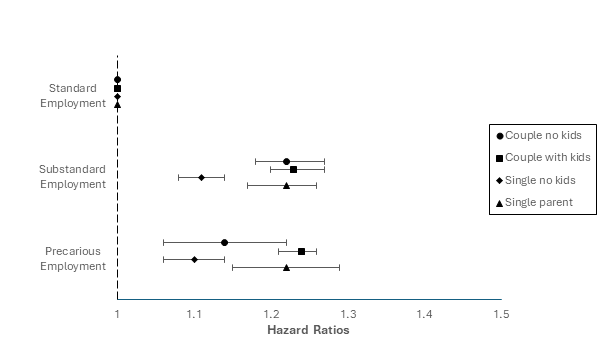


Figure 2: Adjusted hazard ratios for the association between employment conditions and diagnosed mental disorders stratified by household composition.
